# Lung Adenocarcinoma and Peripheral Blood Eosinophilia

**DOI:** 10.7759/cureus.74386

**Published:** 2024-11-25

**Authors:** Margarida I Pereira, Carolina Saca, Mónica Lopes, Inês Eiriz, Andreia Chaves

**Affiliations:** 1 Pulmonology, Hospital Professor Doutor Fernando Fonseca, Lisbon, PRT; 2 Internal Medicine, Hospital Professor Doutor Fernando Fonseca, Lisbon, PRT; 3 Medical Oncology, Hospital Professor Doutor Fernando Fonseca, Lisbon, PRT

**Keywords:** immune-checkpoint inhibitor adverse effects, metastatic non-small cell lung cancer, paraneoplastic syndromes, peripheral eosinophilia, primary lung adenocarcinoma

## Abstract

There are many causes of peripheral blood eosinophilia (PBE), including allergic, infectious, rheumatic, and hematologic disorders. Solid tumor cancers, such as lung cancer, can also cause PBE, and although rare, being diagnosed with PBE in this way is associated with a worse prognosis than for lung cancer patients without PBE. Additionally, some cancer patients develop PBE when receiving treatment with immune checkpoint inhibitors (ICIs). When PBE is caused in this way, the outcome varies. On the one hand, there are reports of severe adverse events, while on the other, ICIs-induced PBE may be a predictive biomarker of better outcomes. This article reports on the case of a 66-year-old male patient with metastatic lung adenocarcinoma presenting with paraneoplastic hypereosinophilia aggravated by pembrolizumab.

## Introduction

Lung cancer remains the most common cause of cancer-related deaths worldwide [1,2]. Some patients with lung cancer present peripheral blood eosinophilia (PBE) (>500 cells/mcL) or even hypereosinophilia (>1500 cells/mcL) [1-6]; however, paraneoplastic eosinophilia (PE) is considered rare in non-hematologic tumors [1,3]. Many patients with lung cancer are now treated with immune checkpoint inhibitors (ICIs), with some developing eosinophilia during treatment [2-4].

## Case presentation

**Figure 1:**
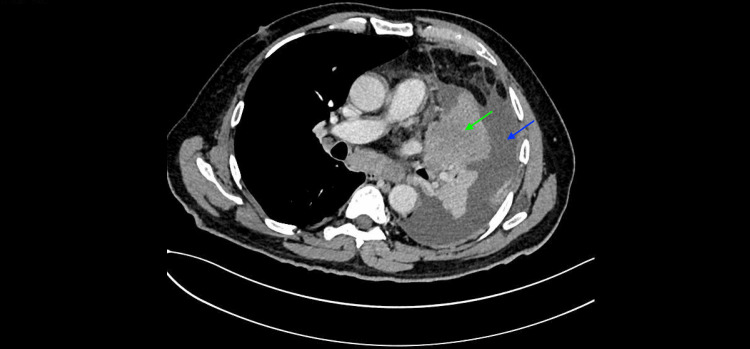
Chest CT scan image The image shows a lung mass (green arrow) in the left superior lobe and left pleural effusion (blue arrow). CT: Computed tomography.

This article reports the case of a 66-year-old male patient who is known to be a former smoker (five pack-years) with a history of allergic rhinitis with nasal polyps (baseline PBE 600 cells/mcL). The patient presented to the emergency department with a four-month history of exertion fatigue and productive cough, denying having any other symptoms. He arrived with a computed tomography (CT) of his chest, showing a lung mass in his left superior lobe, left pleural effusion, micronodules in his right lung, and mediastinal lymphadenopathy (Figure 1).

The patient’s laboratory test results showed neutrophilic leukocytosis and PBE (3500 cells/mcL) (Table 1).

**Table 1:** The patient's laboratory test results at the time of first hospitalization White blood cells (WBC), neutrophils, eosinophils, and C-reactive protein (CRP) trend during the patient’s first hospitalization. The values between parentheses represent reference intervals.

Hospitalization day	WBC (4.000-10.000 cells/mcL)	Neutrophils (1.800-6.900 cells/mcL)	Eosinophils (<600 cells/mcL)	CRP (<0.50 mg/dL)
Day 1	15.900	9.400	3.500	3.16
Day 2	13.600	8.000	2.800	3.25
Day 5	14.700	9.200	2.700	5.70
Day 7	13.800	9.200	2.600	13.06
Day 9	11.700	7.700	1.800	12.63
Day 12	10.800	7.100	2.100	5.43

Additionally, the patient underwent thoracentesis with pleural biopsies, which identified lung adenocarcinoma. His clinical stage was cT3N2M1c (pleural, hepatic, and non-regional lymph node metastases), his expression of programmed death-ligand 1 was 100%, and the driver mutations detection by next-generation sequencing identified MET exon 14 skipping mutation. The investigation excluded infectious, rheumatic, and hematologic disorders as causes of eosinophilia. It was assumed to be paraneoplastic hypereosinophilia.

The patient’s Eastern Cooperative Oncology Group Performance Status (ECOG-PS) score was 1, and a decision was taken to initiate treatment with pembrolizumab monotherapy. Two weeks after the first cycle, the patient returned to the emergency department reporting dyspnea, pleuritic chest pain, a dry cough, and fever. It was also noted that he was hypoxemic at room air. His lab tests showed neutrophilic leukocytosis with PBE (8600 cells/mcL) and an elevated C-reactive protein (Table 2).

**Table 2:** The patient's laboratory test results at the time of second hospitalization White blood cells (WBC), neutrophils, eosinophils, and C-reactive protein (CRP) trend during the patient’s second hospitalization. The values between parentheses represent reference intervals.

Hospitalization day	WBC (4.000-10.000 cells/mcL)	Neutrophils (1.800-6.900 cells/mcL)	Eosinophils (<600 cells/mcL)	CRP (<0.50 mg/dL)
Day 1	21.500	9.500	8.600	9.53
Day 4	20.900	9.400	8.100	11.60
Day 6	28.900	13.500	11.500	4.27
Day 10	23.000	7.500	12.100	5.80
Day 12	20.900	7.200	10.300	3.88
Day 13	19.400	7.000	9.600	-
Day 14 –1^st^ cycle ChT	23.500	10.600	9.100	6.15
Day 17	18.000	17.000	100	2.35
Day 18	14.600	13.300	300	-

**Figure 2:**
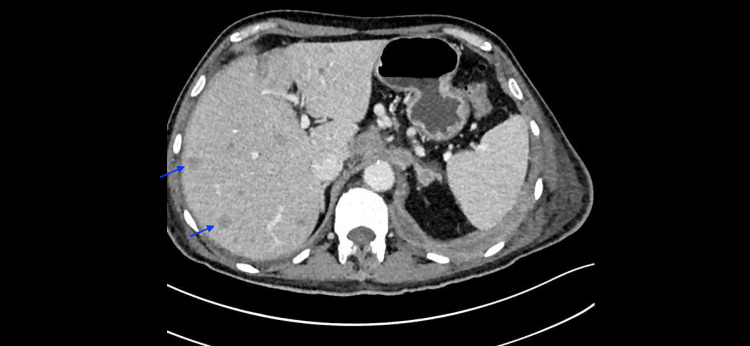
Abdominal CT scan image The image shows liver metastases (blue arrows). CT: Computed tomography.

**Figure 3:**
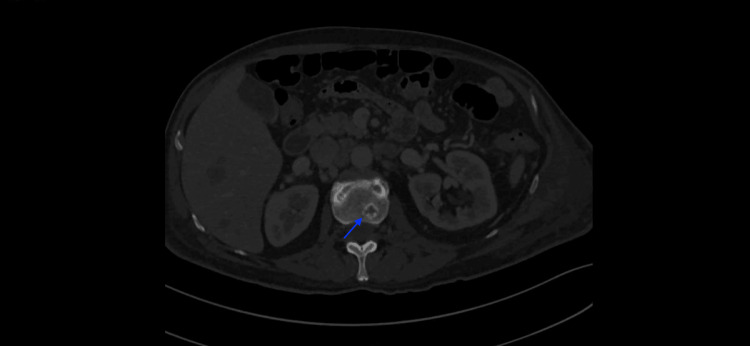
Abdominal CT scan The image shows bone metastases (blue arrow). CT: Computed tomography.

His chest X-ray was suggestive of pneumonia. As a treatment, the patient initiated empirical antibiotic therapy. However, further chest and abdominal CT reassessment documented a progression of the disease, showing the enlargement of previous liver metastases (Figure 2) and de novo adrenal and bone metastases (Figure 3). The patient’s PBE reached a maximum of 12100 cells/mcL (Table 2).

Working under the assumption that pembrolizumab may have contributed to the aggravation of the patient’s hypereosinophilia, it was decided to discontinue treatment with pembrolizumab and to change to chemotherapy with carboplatin/pemetrexed once the complication of infection had been resolved. As expected, the patient’s hypereosinophilia was resolved after the initiation of chemotherapy (Table 2). However, following three cycles of chemotherapy, a reassessment via chest and abdominal CT showed that the disease had progressed. This was accompanied by a new significant increase in the patient’s eosinophil count, corroborating with a paraneoplastic hypereosinophilia diagnosis. Currently, the patient is awaiting approval for treatment with the mesenchymal-epithelial transition factor (MET) inhibitor, tepotinib.

## Discussion

The role of eosinophils in lung cancer is not yet fully understood [1-4]. However, it is believed that eosinophilia results from the tumor’s production of interleukin-5, which is a known main stimulus for eosinophil production in bone marrow [1,3]. When considering lung cancer, the presence of PE has been associated with a more aggressive tumor, extensive metastasis, poor response to therapy, and, consequently, a worse prognosis than for patients without PE [1,3]. It should be noted that PE may not respond to systemic steroid therapy, meaning the treatment should be directed at the tumor itself [1,3].

It has been suggested that eosinophilia may be a predictive biomarker of better outcomes in patients with non-small cell lung cancer treated with ICIs [2,4]. Studies suggest a better overall response rate, disease control rate, and overall survival for patients with eosinophilia during this treatment [2,4]. However, these patients are more likely to experience adverse events, but these are mostly mild and present no reason to discontinue therapy [2]. Nevertheless, it should be noted that there are case reports of severe eosinophilic adverse events induced by ICIs [5,6].

## Conclusions

The significance of PE varies depending on the type of underlying tumor. When discussing non-small cell lung cancer, PE has been associated with a worse prognosis than for patients without PE. This scenario is rare, and as such, it is important to first exclude other causes of eosinophilia, such as allergic, infectious, rheumatic, and hematologic disorders, before making a diagnosis.

There exist a number of hypotheses concerning the role of eosinophils in cancer pathophysiology and ICIs treatment, however, further studies with larger samples or more case reports are needed to improve our understanding of this issue.
